# Mediterranean Diet on Development and Progression of Age-Related Macular Degeneration: Systematic Review and Meta-Analysis of Observational Studies

**DOI:** 10.3390/nu17061037

**Published:** 2025-03-15

**Authors:** Pedro Marques-Couto, Inês Coelho-Costa, Renato Ferreira-da-Silva, José Paulo Andrade, Ângela Carneiro

**Affiliations:** 1 Ophthalmology Department, Local Health Unit of São João, 4099-450 Porto, Portugal; ines.coelho.costa@ulssjoao.min-saude.pt (I.C.-C.); angela.carneiro@ulssjoao.min-saude.pt (Â.C.); 2 Porto Pharmacovigilance Centre, Faculty of Medicine, University of Porto, 4200-450 Porto, Portugal; rsilva@med.up.pt; 3 RISE-Health, Department of Community Medicine, Information and Health Decision Sciences, Faculty of Medicine, University of Porto, 4200-450 Porto, Portugal; 4 RISE-Health, Biomedicine Department—Unity of Anatomy, Faculty of Medicine, University of Porto, 4200-450 Porto, Portugal; jandrade@med.up.pt; 5 RISE-Health, Surgery and Physiology Department, Faculty of Medicine, University of Porto, 4200-450 Porto, Portugal

**Keywords:** diet, mediterranean, macular degeneration, risk factors, evidence-based medicine, epidemiology, nutritional status

## Abstract

Introduction: Age-related macular degeneration (AMD) is a leading cause of vision impairment. A Mediterranean diet (MD) has been suggested to provide protective effects against AMD development and progression due to its antioxidant and anti-inflammatory properties. However, inconsistencies in findings across observational studies have been reported. This systematic review and meta-analysis aim to synthesize the existing evidence on the relationship between adherence to the MD and AMD development or progression. Methods: A systematic search was conducted using MEDLINE (via PubMed), Web of Science, and SCOPUS, following PRISMA guidelines. Observational studies assessing MD adherence in relation to AMD risk or progression were included. Meta-analyses were performed separately for each study design using odds ratios (ORs) for cross-sectional and case–control studies and hazard ratios (HRs) for prospective cohort studies. Heterogeneity was assessed using the *I*^2^ statistic, and publication bias was evaluated via funnel plots. Results: Eight studies were included: two cross-sectional, three case–control, and three prospective cohort studies. The meta-analysis of cross-sectional studies (pooled OR = 0.96; 95% CI: 0.83–1.11; *p* = 0.6243; *I*^2^ = 0%) found no significant association between MD adherence and AMD. However, the weight distribution was highly imbalanced, limiting interpretability. Meta-analyses of case–control and prospective cohort studies demonstrated a significant protective effect of MD adherence: case–control studies showed a 34% reduction in the odds of AMD progression (OR = 0.66; 95% CI: 0.54–0.81; *p* < 0.0001; *I*^2^ = 41.5%), while prospective cohort studies indicated a 23% reduced risk of AMD progression (HR = 0.77; 95% CI: 0.67–0.88; *p* < 0.0001; *I*^2^ = 0%). Conclusions: This systematic review and meta-analysis suggest an inverse association between adherence to the MD and AMD progression, particularly in case–control and prospective cohort studies. Despite the small number of included studies, these findings highlight the potential role of diet in AMD management. Future research should focus on larger, well-controlled prospective studies with standardized dietary assessments. Key Points: 1. Higher adherence to the MD is associated with a reduced risk of AMD progression, with meta-analyses of case–control and prospective cohort studies showing 34% lower odds (OR = 0.66) and 23% reduced risk (HR = 0.77) of disease progression, respectively. 2. No significant association was found in cross-sectional studies (OR = 0.96; 95% CI: 0.83–1.11), likely due to methodological limitations and the challenge of establishing a temporal relationship between diet and AMD progression. 3. Despite the limited number of studies, findings suggest a potential role of diet in AMD management. Future research should prioritize larger, well-controlled prospective studies with standardized dietary assessments.

## 1. Introduction

Age-related macular degeneration (AMD) is a leading cause of severe vision impairment in older adults, affecting approximately 196 million people globally, a number projected to rise to 288 million by 2040 [1,2]. It is marked by the progressive accumulation of extracellular deposits (drusen) in the outer retina, leading to chronic oxidative stress, inflammation, and the disruption of photoreceptor function. Over time, these changes result in two distinct advanced forms: geographic atrophy (GA), marked by progressive loss of retinal pigmented epithelium (RPE) and photoreceptors, and neovascular AMD (nAMD), characterized by abnormal macular neovascularization followed by vascular leakage and fibrosis. While GA leads to gradual central scotomas, nAMD can be associated with rapid and severe visual loss due to hemorrhage and fluid accumulation [3].

The condition is complex and multifactorial, influenced by a combination of genetic, environmental, and lifestyle factors, with older age, cigarette smoking, and genetic predisposition being the primary risk factors [4,5]. Lifestyle factors, particularly diet, have been recognized as modifiable risk factors for AMD [6,7,8].

The Mediterranean diet (MD), a nutritional pattern characterized by a high intake of fruits, vegetables, whole grains, legumes, nuts, and olive oil, along with moderate consumption of fish and poultry, has gained attention for its potential role in AMD prevention [9]. The MD is rich in antioxidants, anti-inflammatory compounds, and essential nutrients such as omega-3 fatty acids, lutein, zeaxanthin, and polyphenols, which may mitigate oxidative stress and inflammation, two key pathways implicated in AMD pathogenesis [10,11,12].

Recent evidence suggests that adherence to the MD may influence AMD progression. Observational studies have demonstrated that higher adherence to the MD is associated with reduced risk of AMD development and slower progression to advanced stages [13,14]. However, inconsistencies in findings across studies underscore the need for further research to clarify these associations.

This systematic review aims to critically evaluate and synthesize existing evidence on the relationship between MD adherence and AMD development or progression to enhance our understanding of the potential role of dietary interventions in AMD prevention and management.

## 2. Methods

Our study adhered to established protocols for conducting and reporting systematic reviews concerning public health interventions in healthcare, as outlined by the Cochrane Handbook for Systematic Reviews of Interventions [15] and the Preferred Reporting Items for Systematic Reviews and Meta-Analyses (PRISMA) guidelines [16]. The protocol for this review was registered in PROSPERO under the identifier CRD42024584279.

### 2.1. Eligibility Criteria

Observational studies (including prospective and retrospective cohort studies, cross-sectional studies, and case–control studies) that included patients either at risk of or already diagnosed with AMD and examined the influence of MD intake on disease development and/or progression were included. Development of AMD was characterized by classification according to the AREDS classification system, specifically category 1, i.e., absence or presence of small drusen (<63 µm) within two disk diameters of the macula center and no pigment abnormalities [17]. Progression of AMD was defined as the transition between or within AREDS categories [17]. Adherence to the MD was defined as the consistent and substantial adherence to its main components, including high consumption of fruits, vegetables, legumes, whole grains, fish, and olive oil, with moderate wine consumption. Studies focusing solely on isolated micronutrients or individual food components and those assessing dietary patterns other than the MD were excluded.

Systematic reviews, literature reviews, commentaries, protocols, and studies lacking primary data or quantitative analysis were also excluded. No restrictions were applied regarding participants’ age, sex, ophthalmic condition, or comorbidities. Additionally, no restrictions were placed on publication date, language, or geographic region.

### 2.2. Information Sources and Search Strategy

A systematic literature search was conducted across MEDLINE (via PubMed), Web of Science, and SCOPUS to identify relevant studies. The complete search strategy is detailed in Table S1. No restrictions were placed on publication date or language, with translations or assessments performed by language-proficient individuals when necessary for non-English articles. Beyond database searches, we reviewed the reference lists of all included studies to identify additional relevant research, including unpublished or in-press works. Expert consultations were also carried out to uncover further unpublished materials until no new sources emerged.

### 2.3. Study Selection and Data Extraction

Study selection was carried out independently by two reviewers, initially through title/abstract screening (I.C.-C. and P.M.-C.) and subsequently through full-text reading (I.C.-C. and P.M.-C.). A third reviewer (J.P.A.) resolved disagreements at each phase. Endnote was used to initially remove duplicates and manage the bibliography of the selected literature, and the Rayyan app (http://rayyan.qcri.org, accessed on 16 February 2025) was used to expedite the screening of abstracts, further eliminate duplicates, and facilitate the selection and organization of full-text articles for inclusion.

Two reviewers (I.C.-C. and P.M.-C.) independently extracted data from the included studies using a custom-designed internal online form. The extracted variables comprised authorship, publication year, country, study design, inclusion period, follow-up duration, eligible population, participant demographics (age and sex), sample size, exposure and outcome assessments, and variables used for outcome comparisons.

Any disagreements arising during the review process were settled by a third reviewer (R.F.-d.-S.), guaranteeing a consistent and rigorous approach. Reviewer agreement at the selection stage was evaluated using the kappa coefficient.

### 2.4. Risk-of-Bias (Quality) Assessment

Two researchers (P.M.-C. and I.C.-C.) independently evaluated the risk of bias in each included primary study using the National Institutes of Health (NIH) Quality Assessment Tools, chosen based on the specific observational study design [18]. These tools evaluate key methodological domains, including research question clarity, population definition, sample size justification, exposure and outcome assessment, blinding, follow-up rates, and adjustment for confounders. Each study received an overall quality rating of “good”, “fair”, or “poor” based on adherence to these criteria and the resulting risk of bias. Studies rated as “good” met most or all quality criteria and presented a minimal risk of bias, studies rated as “fair” met several quality criteria but presented a moderate risk of bias, and studies rated as “poor” met few or none of the quality criteria and presented a high risk of bias. Studies were classified as “good” if they had up to three high-risk ratings, provided that key methodological domains, such as exposure and outcome assessment and adjustment for confounders, were rated as low risk. Studies with four to five high-risk ratings were classified as “fair”, while those with six or more high-risk ratings were considered “poor”.

To ensure consistency and accuracy in the assessment process, discrepancies between reviewers were resolved through discussion and, when necessary, by consulting a third assessor. The overall quality ratings were used to inform the interpretation of results and the strength of evidence derived from the included studies.

The Robvis tool (Version: 0.3.0) was employed to visually represent the risk of bias assessments and generate summary plots for transparency and clarity in reporting [19].

### 2.5. Quantitative Synthesis

A meta-analysis was conducted separately for cross-sectional, case–control, and prospective cohort studies due to their methodological differences and distinct effect measures. For both cross-sectional and case–control studies, odds ratios (ORs) with corresponding 95% confidence intervals (95% CIs) were used, while for prospective cohort studies, hazard ratios (HRs) with 95% CIs were considered.

Both common-effect and random-effects models were considered based on the level of heterogeneity (*I*^2^) and the methodological and clinical characteristics of the included studies. Between-study variability was quantified using the *I*^2^ statistic and the τ^2^ parameter, the latter estimated through the Restricted Maximum Likelihood (REML) approach. Heterogeneity was assessed using the Q-Cochran test (*p*-value) and the *I*^2^ statistic, with *I*^2^ ≥ 40% and *p*-values < 0.10 considered indicative of severe and significant heterogeneity, respectively.

Heterogeneity sources were evaluated through a leave-one-out sensitivity analysis, which was applied exclusively to study groups with severe/significant heterogeneity (*I*^2^ ≥ 40%), where each study was sequentially removed to evaluate its influence on the overall pooled estimate. Baujat plots were used to detect studies disproportionately contributing to heterogeneity. Publication bias was assessed via funnel plots, and results were visualized using forest plots.

Statistical analyses were performed in R (version 2023.06.1+524, R Foundation for Statistical Computing, Vienna, Austria) utilizing the meta and metafor packages.

## 3. Results

### 3.1. Search Results

**Figure 1:**
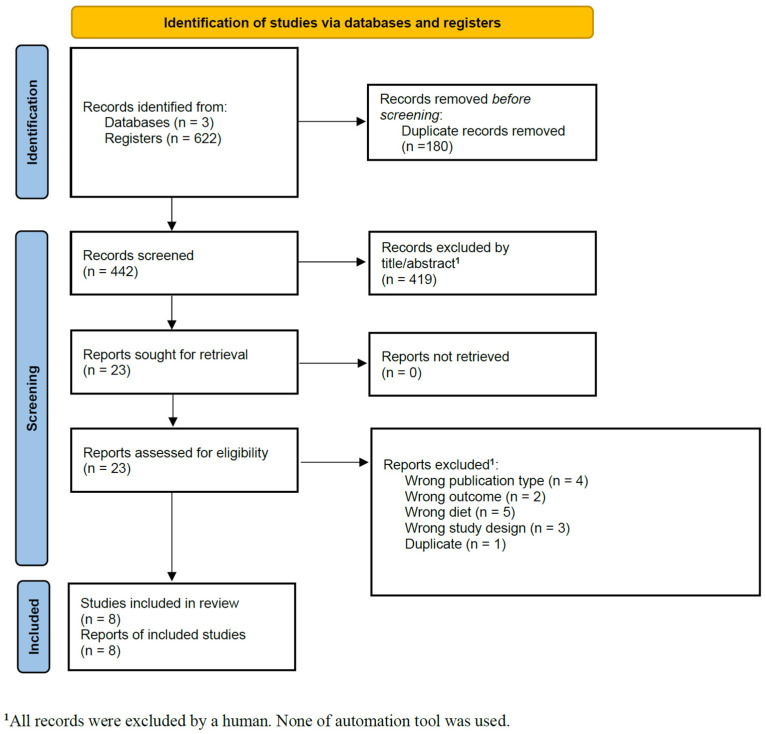
PRISMA flow diagram for primary study selection.

The database search identified 622 records, from which 180 duplicates were removed. The remaining 442 studies underwent title and abstract screening, leading to the exclusion of 419 studies. A total of 23 full-text articles were assessed for eligibility. Of these, four were excluded due to incorrect publication type [20,21,22,23], two for evaluating the wrong outcome [24,25], five for assessing diets other than the MD [26,27,28,29,30], three for inappropriate study design [6,31,32], and one was a duplicate [33] (Table S2). In the end, eight studies fulfilled the inclusion criteria and were incorporated into the systematic review and meta-analysis [13,14,33,34,35,36,37,38]. At the end of the screening phase, the kappa coefficient was 0.609 (95% CI: 0.487–0.730f). Upon completing the selection by full-text reading, the kappa coefficient reached 1.0 (95% CI: 1.0–1.0). The study selection process is illustrated in Figure 1.

### 3.2. Description of Studies

The eight studies included comprised two cross-sectional [13,34], three case–control studies [35,36,37], and three prospective cohort studies [14,33,38] conducted in Portugal [35,36,37], Greece [13], France [34], Italy [34], Spain [34], Norway [34], Estonia [34], the United Kingdom [34], and the United States [22,38].

The cross-sectional studies assessed the association between MD adherence and AMD prevalence, while case–control studies compared dietary patterns between AMD cases and controls, adjusting for age, sex, smoking, body mass index (BMI), and other lifestyle factors. The prospective cohort studies examined the long-term impact of MD adherence on AMD progression, with follow-up periods ranging from several years to over a decade.

Sample sizes varied from 164 to 4996 participants [13,33], with ages ranging from 55 to 80 years. Sex distribution varied across studies, with the proportion of female participants ranging from 50.0% to 65.9%. All studies had a slightly higher representation of female patients, reflecting the greater prevalence of AMD in this population. AMD classification systems differed among studies, including AREDS2, International Classification for Age-Related Maculopathy, Rotterdam, CARMS, Modified Wisconsin, and International AMD Classification. Severity ranged from no AMD to advanced stages, with one study applying age-specific criteria for controls [37].

The core components of the MD were consistent across the different trials—each study emphasized high intakes of fruits, vegetables, legumes, whole grains, fish, and olive oil, along with moderate alcohol consumption and low red meat intake. However, there were minor differences in how adherence was quantified. MD adherence was assessed using validated Food Frequency Questionnaires (FFQs), with most studies calculating an MD score (MediScore or aMED score). In some studies, adherence was categorized into low, medium, and high [14,33,35], while others used quantile-based classifications [34,36,37,38].

A detailed summary of study characteristics and AMD classification systems is presented in Table 1, while Table 2 provides an overview of exposure assessments, AMD outcomes, comparator groups, and effect estimates.

### 3.3. Main Findings and Meta-Analysis

#### 3.3.1. Cross-Sectional Studies

In a large multicenter study, Hogg et al. (2017) [34] assessed dietary intake using semi-quantitative Food Frequency Questionnaires (FFQs) and classified AMD based on the International Classification of Age-Related Maculopathy. Gourgouli et al. (2011) [13], a smaller study from Greece, used a similar dietary assessment method but defined AMD progression based on visual acuity deterioration and anatomical changes.

Gourgouli et al. (2011) [13] reported no significant association between MD adherence and AMD (OR = 2.43; 95% CI: 0.31–19.18; *p* = 0.397). In Hogg et al. (2017) [34], MD adherence showed no significant trend for early AMD (OR = 0.96; 95% CI: 0.83–1.11; *p*-trend = 0.9) but suggested a modest protective effect for large drusen (OR = 0.80; 95% CI: 0.65–0.98; *p*-trend = 0.1) and neovascular AMD (OR = 0.53; 95% CI: 0.27–1.04; *p*-trend = 0.01), though the results were not statistically significant.

**Figure 2:**
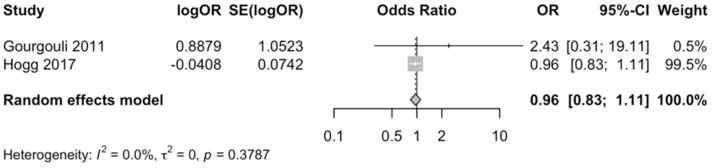
Forest plot of the meta-analysis of cross-sectional studies assessing the association between Mediterranean diet adherence and disease progression [13,34].

The meta-analysis of these studies yielded a pooled OR of 0.96 (95% CI: 0.83–1.11; *p* = 0.6243; *I*^2^ = 0%), indicating no significant association between MD adherence and AMD (Figure 2). The weight distribution was highly imbalanced, with Hogg et al. (2017) [34] contributing 99.5% to the pooled estimate, while Gourgouli et al. (2011) [13] had a minimal impact (0.5%). Baujat plot analysis confirmed that Hogg et al. (2017) [34] had the greatest influence on the pooled effect size but did not contribute meaningfully to heterogeneity (Figure S1).

#### 3.3.2. Case–Control Studies

Nunes et al. (2018) [35] and Raimundo et al. (2018) [36], both case–control studies conducted in Portugal, assessed dietary adherence using a validated 86-item FFQ and classified AMD based on the Rotterdam Classification. Barreto et al. (2023) [37], also a case–control study from Portugal, applied a similar dietary scoring system but had broader inclusion criteria, considering individuals with early AMD as controls if they met specific age-related thresholds.

All three case–control studies reported an inverse association between MD adherence and AMD prevalence. Nunes et al. (2018) [35] found a significant association, with a 34% reduction in AMD prevalence among those with high MD adherence (OR = 0.73; 95% CI: 0.58–0.93; *p* = 0.009). Similarly, Raimundo et al. (2018) [36] reported a 37% reduction in AMD prevalence (OR = 0.63; 95% CI: 0.41–0.98; *p* = 0.043). Barreto et al. (2023) [37] showed the strongest effect, with 59% lower odds of AMD in individuals with high MD adherence (OR = 0.41; 95% CI: 0.23–0.73; *p* = 0.002). These results support a protective association between MD adherence and lower AMD prevalence, with consistent findings across all case–control studies.

**Figure 3:**
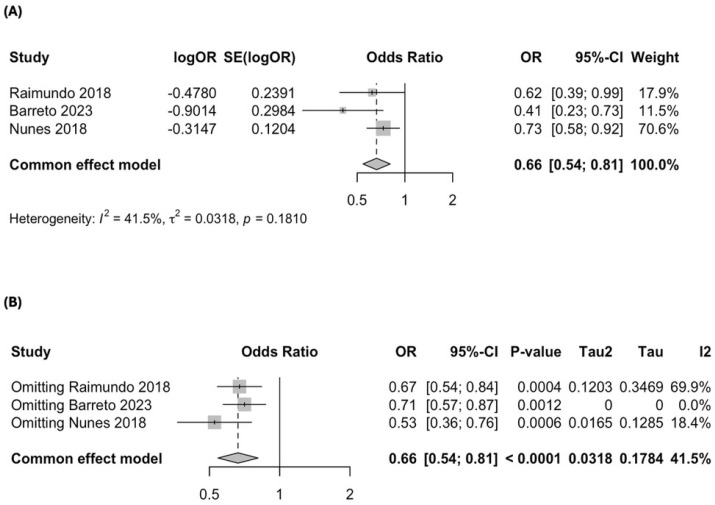
Forest plot of the meta-analysis of case–control studies assessing the association between Mediterranean diet adherence and disease progression: (**A**) forest plot including all case–control studies; (**B**) leave-one-out sensitivity analysis evaluating the influence of individual studies on the overall pooled estimate [35,36,37].

The meta-analysis of these studies found a significant association between MD adherence and lower odds of AMD, with a pooled OR of 0.66 (95% CI: 0.54–0.81; *p* < 0.0001; *I*^2^ = 41.5%), corresponding to a 34% reduction in AMD risk (Figure 3A). A leave-one-out sensitivity analysis showed that removing Barreto et al. (2023) [37] eliminated heterogeneity (*I*^2^ = 0%), but all were retained given the limited number of studies (Figure 3B). The Baujat plot identified Barreto et al. (2023) [37] as the study contributing the most to heterogeneity, whereas Nunes et al. (2018) [35] had the most significant influence on the overall estimate (Figure S2).

#### 3.3.3. Prospective Cohort Studies

Three prospective cohort studies were identified. Merle et al. (2015) [14] assessed dietary patterns in the AREDS cohort using a self-administered 90-item FFQ and classified AMD progression with the CARMS system. Merle et al. (2019) [33] utilized two European cohorts (Rotterdam Study and Alienor Study), applying different AMD classification systems and dietary assessment tools. Merle et al. (2020) [38], another AREDS-based study, investigated how MD adherence influenced maximum drusen size progression over time.

A protective association between MD adherence and AMD progression was reported by all prospective cohort studies. Merle et al. (2015) [14] found no significant association when comparing high versus low MD adherence (HR = 0.92; 95% CI: 0.78–1.07), but individuals with medium adherence had a lower risk of AMD progression (HR = 0.74; 95% CI: 0.61–0.91). Merle et al. (2019) [33] reported that high MD adherence was associated with a significantly lower risk of advanced AMD (HR = 0.59; 95% CI: 0.37–0.95), while medium adherence showed a borderline significant effect (HR = 0.70; 95% CI: 0.49–1.01). Merle et al. (2020) [38] found that moderate-to-high MD adherence (≥4) was linked to a lower risk of progression in maximum drusen size (HR = 0.83; 95% CI: 0.68–0.99; *p* = 0.049). These findings suggest a dose–response trend, with higher MD adherence associated with a lower risk of AMD progression.

**Figure 4:**
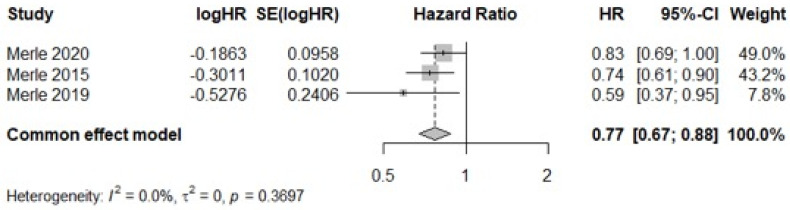
Forest plot of the meta-analysis of prospective cohort studies assessing the association between Mediterranean diet adherence and disease progression [14,33,38].

The pooled meta-analysis of these studies also demonstrated a significant protective effect of the MD, with a hazard ratio (HR) of 0.77 (95% CI: 0.67–0.88; *p* < 0.0001; *I*^2^ = 0%), corresponding to a 23% reduction in AMD progression risk (Figure 4). Sensitivity analysis confirmed that all studies contributed proportionally to the pooled estimate, and the Baujat plot showed no single study exerted undue influence on the findings (Figure S3).

### 3.4. Studies Quality and Publication Bias

**Figure 5:**
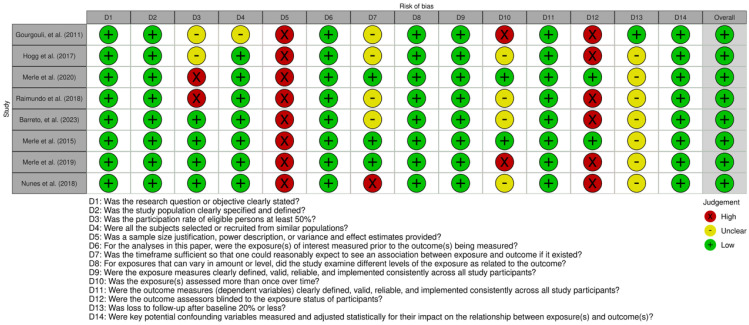
Global traffic-light plot for risk of bias based on NIH Quality Assessment Tools for observational studies [13,14,33,34,35,36,37,38].

**Figure 6:**
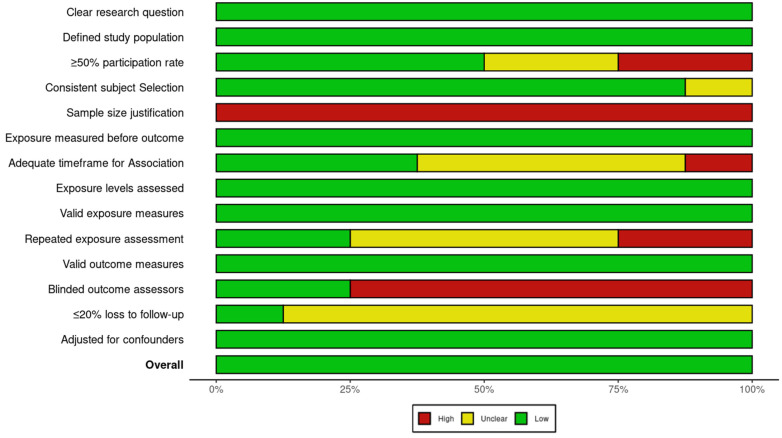
Global summary plot for risk of bias based on NIH Quality Assessment Tools for observational studies.

The risk of bias assessment, summarized in Figure 5 and Figure 6, indicates that most studies had a low to moderate risk of bias, with prospective cohort studies showing the highest methodological quality. While study populations and outcome assessments were generally well defined, sample size justification was lacking, and participation rates below 50% in some studies introduced potential selection bias. The blinding of outcome assessors was inconsistent, which may have influenced AMD classification, and dietary adherence was typically assessed only once, limiting accuracy. Loss to follow-up exceeded 20% in some prospective studies, though most adjusted for key confounders, strengthening validity.

Funnel plot analysis (Figures S4–S6) showed no apparent publication bias in prospective cohort studies, while case–control studies displayed some asymmetry, suggesting possible bias or variability in effect estimates. The small number of cross-sectional studies limited the funnel plot interpretation. Despite these concerns, prospective cohort studies provided the strongest evidence for a protective effect of MD on AMD progression.

## 4. Discussion

This systematic review and meta-analysis synthesized the evidence from eight studies investigating the association between adherence to the MD and the progression of AMD, stratifying the analysis by study design due to their distinct effect measures and methodological characteristics. The findings indicate an inverse association between MD adherence and AMD progression, with a 34% reduction in disease progression risk observed in case–control studies and a 23% reduction in prospective cohort studies. In contrast, cross-sectional studies did not support this association, possibly due to inherent design limitations and the small number of studies available.

The Mediterranean diet (MD) is rich in polyunsaturated fatty acids (PUFAs) ω-3, vitamin C, vitamin E, and carotenoids (lutein and zeaxanthin)—micronutrients with well-established antioxidant and neuroprotective properties [39]. In the retina, PUFAs—particularly eicosa-pentaenoic acid (EPA, C20:5), docosahexaenoic acid (DHA, C22:6), and α-linolenic acid (ALA, C18:3)—are integral components of photoreceptor outer segments, playing a crucial role in maintaining membrane fluidity, protecting photoreceptors from oxidative damage, and preventing apoptosis [40]. Vitamin C supports retinal pigment epithelium (RPE) metabolism and enhances inner retinal function [41], while vitamin E is known to decrease phototoxic damage to the photoreceptor outer segments because of its ability to decrease lipid peroxidation [42]. Additionally, lutein and zeaxanthin contribute to mitochondrial stability in the RPE and reduce oxidative stress, further supporting retinal health [43,44].

Previous trials such as AREDS [17] and AREDS2 [11] have demonstrated the benefits of macular xanthophyll (lutein and zeaxanthin) supplementation in slowing AMD progression among patients in AREDS categories 3 and 4; however, AREDS2 found no significant benefit of omega-3 fatty acid supplementation, though it may still be beneficial when obtained through diet (e.g., MD) but not as proven supplements for AMD [45]. However, many commercially available formulations exhibit substantial variation in nutrient dosages [46,47], and long-term supplementation may present challenges related to cost and adherence [48]. Given these challenges, exploring dietary strategies for both AMD prevention and progression remains essential. The MD, rich in fruits, vegetables, legumes, whole grains, fish, and olive oil, while low in red meat and with moderate alcohol consumption [36], has shown promise in relation to AMD [49]; however, a comprehensive quantitative assessment of the association between MD and reduced AMD risk is needed. Therefore, this review rigorously evaluated the existing evidence and quantified the association between adherence to the MD and the risk of AMD development and progression.

Our meta-analysis of cross-sectional studies showed no significant association between adherence to the MD and AMD progression; however, this result is limited by the small number of included studies and the imbalanced weight distribution, with one study [34] contributing to the majority of the influence on the pooled estimate. While there was no heterogeneity, the lack of studies and the disproportionate weight of a single study compromise the robustness of this conclusion, preventing generalizations.

In contrast, the meta-analyses of case–control and prospective cohort studies revealed significant associations between higher adherence to the MD and a lower likelihood of AMD progression. In case–control studies, we observed a 34% reduction in the odds of disease progression, while in prospective cohort studies, there was a 23% reduction in the risk of progression. The heterogeneity observed in the case–control studies was explored through sensitivity analysis, which identified the study by Barreto et al. (2023) [37] as the main contributor to the heterogeneity. Removing this study eliminated the heterogeneity, but due to the limited number of studies, we retained all three in the main analysis, recognizing the importance of considering the totality of available evidence. It is well established that the AREDS formula only demonstrated a protective effect against the exudative form [45]. Therefore, future studies should focus on evaluating whether the Mediterranean diet has a distinct impact on the development of different forms of the disease, exploring underlying mechanisms and investigating whether its influence varies depending on the AMD subtype.

The discrepancy in results between the different study designs raises important questions about interpreting the relationship between the MD and AMD. The absence of an association in cross-sectional studies may be attributed to their observational nature and the difficulty in establishing the temporality between exposure (diet) and outcome (AMD progression). Cross-sectional and case–control studies assess diet and disease progression simultaneously, making it difficult to determine whether the MD influences AMD progression or whether AMD progression influences adherence to the diet. On the other hand, prospective cohort studies, which assess exposure (diet) before or over time in relation to the outcome (AMD progression), offer greater capacity to infer causal relationships. Our results, consistent with a protective effect of the MD on AMD progression in cohort and case–control studies, suggest that diet may influence the course of the disease. This finding aligns with the established understanding of AMD pathogenesis, where oxidative stress and inflammation play critical roles [50]. The MD, rich in antioxidants, anti-inflammatory compounds, and essential nutrients [51,52], may mitigate these processes, thereby potentially slowing the degenerative cascade observed in AMD.

It is crucial to acknowledge the limitations of this review. Firstly, the limited number of included studies not only affects the statistical power of our findings but also impacts the reliability of effect estimates. With fewer studies, the risk of random variations influencing the results increases, and the potential for publication bias cannot be overlooked. Secondly, the heterogeneity observed in some study groups, despite being explored, and the potential influence of confounding factors not uniformly assessed among the studies should also be considered. Thirdly, the lack of standardization in assessing adherence to the MD among the studies may have contributed to the variability in effect estimates. Fourthly, the assessment of dietary adherence mostly relied on FFQs, which are subject to recall bias and may not accurately reflect long-term dietary patterns [53]. Most studies assessed dietary adherence only once, which limits our understanding of how changes in dietary habits over time may influence AMD risk. Finally, while we did not apply language restrictions, it is important to note that the studies included in this review were primarily conducted in European and North American populations, reflecting the geographic distribution of available research on this topic. This may limit the generalizability of our findings to other regions where dietary patterns and genetic backgrounds differ, highlighting the need for future studies assessing the impact of the MD on AMD in more diverse populations [54,55]. As strengths, the present review benefits from a rigorous methodology, adhering to established guidelines (Cochrane and PRISMA) and a pre-registered protocol. The comprehensive search strategy across multiple databases minimized the risk of publication bias. Furthermore, using the NIH Quality Assessment Tools allowed for a systematic evaluation of the risk of bias in the included studies.

The systematic review and meta-analysis showed a potential protective effect of the MD against AMD, particularly in terms of slowing its progression, as found in prospective cohort and case–control studies, although the evidence for preventing its initial development is less conclusive. However, the small number of studies, especially in the cross-sectional group, warrants caution in interpreting these findings. Future research with larger sample sizes, incorporating standardized AMD classification systems, standardized dietary assessment methods, and longer follow-up periods is needed to confirm these findings and elucidate the specific mechanisms by which the MD may influence the development and progression of AMD. Investigating the synergistic effects of combining the MD with other lifestyle modifications, such as smoking cessation and increased physical activity [56,57], may also yield valuable insights.

## 5. Conclusions

In conclusion, the present findings underscore the potential of dietary interventions in managing AMD progression, reinforcing that promoting adherence to the MD may be a valuable strategy for reducing the burden of this chronic and debilitating disease [58].

## Figures and Tables

**Table 1:** Description of primary studies included in the systematic review, organized by type of study.

Study (Author; Year)	Study Characteristics				Patient Characteristics			
	Location	Study Design	Inclusion Period; Follow-Up Period	Eligible Population Age; Sex ^a^	Patient Disease Stage ^b^ (Classification)	Sample Size	Age Range in Years [Mean (SD) or Median (IQR) *]	Male (n, %)
**Cross-sectional studies**								
Gourgouli, et al. (2011) [13]	Greece	Cross-sectional	2 years; NA	>55 years; both	Early or intermediate AMD (AREDS2 classification)	164	73 (7.4) *	52 (31.7%)
Hogg et al. (2017) [34]	Norway, Estonia, United Kingdom, France, Italy, Greece, Spain	Cross-sectional, within the EUREYE study (cross-sectional study with retrospective and current exposure measurements)	2001–2002; NA	>65 years; both	Any stage AMD (International Classification System for Age-Related Maculopathy)	4753	73.2 (5.6)	45%
**Case–control studies**								
Nunes et al. (2018) [35]	Portugal	Nested case–control study within the “Epidemiologic Study of the Prevalence of Age-Related Macular Degeneration in Portugal: The Coimbra Eye Study” (cross-sectional)	12 months; NA	>55 years; both	Cases: stage 1–4 AMD Controls: stage 0 AMD (Rotterdam Classification)	1992 (768 cases; 1224 controls)	Cases: 71.6 (7.7) Controls: 70.6 (7.0)	Cases: 323 (42.1%) Controls: 556 (45.4%)
Raimundo et al. (2018) [36]	Portugal	Nested case–control study within the “Epidemiologic Study of the Prevalence of Age-Related Macular Degeneration in Portugal: The Coimbra Eye Study” (cross-sectional)	NR; NA	≥55 years; both	Cases: stage 1–4 AMD Controls: stage 0 AMD (Rotterdam Classification)	883 (434 cases; 449 controls)	Cases: 69.7 (7.9) Controls: 69.0 (7.5)	Cases: 187 (43.1%) Controls: 198 (44.1%)
Barreto et al. (2023) [37]	Portugal	Nested case–control study within the “AMD Incidence Study” and the “Lifestyle and Food Habits Questionnaire in the Portuguese Population Aged 55 or More” (both cross-sectional)	2016–2017; NA	≥55 years; both	Cases: stage 2–4 AMD Controls: stage 0 AMD and >60 years or stage 1 AMD and >70 years (Rotterdam Classification)	612 (161 cases; 451 controls)	Cases: 74.8 (6.8) Controls: 71.8 (6.4)	Cases: 60 (37.3%) Controls: 200 (44.3%)
**Prospective cohort studies**								
Merle et al. (2015) [14]	United States	Prospective cohort within AREDS (RCT)	1992–1998; 13 years	55–80; both	Stage 0–3 AMD (CARMS system)	2525 (4663 eyes)	NR	NR
Merle et al. (2019) [33]	Europe	Prospective cohort study of the Rotterdam Study I (RS-I) and Antioxydants, Lipides Essentiels, Nutrition et maladies Oculaires (Alienor) study populations, part of the EYE-RISK project	RS-I: NR; 1990–2011; Alienor: NR; 2006–2012	RS-I: ≥55 years; both Alienor: ≥73 year; both	No AMD or early AMD (modificated Wiconsin Age-Related System for RS-I; Internation Classification for Alienor)	4996 (4446 from RS-I and 550 from Alienor)	NR	NR
Merle et al. (2020) [38]	United States	Prospective cohort within AREDS (RCT)	1992–1998; 13 years	55–80; both	Eyes without drusen or with drusen but without advanced AMD or drusen ≥ 125 µm	1838 (3023 eyes)	NR	1328 (43.9%)

NA: not applicable; NR: not reported; ^a^ F: female; M: male. ^b^ This variable refers to whether participants are at risk of developing age-related macular degeneration (AMD) or have an established diagnosis of the disease. This distinction helps differentiate studies focused on prevention from those analyzing disease progression or treatment. The primary studies were sequentially arranged in the table based on (i) type of study and (ii) year of publication, from oldest to most recent. “*” is used in order to point that the age is reported in median (IQR).

**Table 2:** Overview of exposure, outcomes, and main results from the included primary studies.

Study (Author; Year)	Exposure Assessment	Outcome Assessment	Comparator Group and Statistical Approach	Effect Measure (OR or HR, 95%CI); *p*-Value
**Cross-sectional studies**				
Gourgouli, et al. (2011) [13]	FFQ about the 12 previous months was assessed by a semi-quantitative FFQ MDScore was calculated	AMD progression considered when patients had deterioration in visual acuity and/or anatomical changes	Comparator group: patients without supplement intake and low MD adherence Patients were divided into 4 groups: (1) high adherence/supplement intake, (2) high adherence/no supplement intake, (3) low adherence/supplement intake, (4) low adherence/no supplement intake Logistic regression (unadjusted and adjusted for age, sex, BMI, and smoking)	*Unadjusted OR* = 2.17 (0.30–15.71); 0.444 *Adjusted OR* = 2.43 (0.31–19.18); 0.397
Hogg et al. (2017) [34]	Semiquantitative FFQ (130 foods) tailored to each country MDScore was calculated	AMD graded according to the ICS for Age-Related Maculopathy	Patients were divided into 4 MDScore groups: Q1 ≤ 4, Q2 = 5, Q3 = 6, and Q4 ≥ 6 Association with all early AMD, large Drusen and nvAMD was assessed (unadjusted and adjusted for age, sex, country, education, smoking habits, drinking habits, self-reported history of cardiovascular disease, aspirin consumption, diabetes, and BMI—OR) Q1 was defined as reference	***ALL EARLY AMD*** *Unadjusted OR* Q2: OR = 0.99 (0.92–1.07); Q3: OR = 0.98 (0.89–1.08); Q4: OR = 0.94 (0.85–1.03); *p* trend = 0.4 *Adjusted OR* Q2: OR = 1.01 (0.91–1.12); Q3: OR = 1.01 (0.90–1.14); Q4: OR = 0.96 (0.83–1.11); *p* trend = 0.9 ***LARGE DRUSEN*** *Unadjusted OR* Q2: OR = 0.96 (0.83–1.11); Q3: OR = 0.89 (0.70–1.12): Q4: OR = 0.79 (0.65–0.97); *p* trend = 0.05 *Adjusted OR* Q2: OR = 0.99 (0.80–1.21); Q3: OR = 0.90 (0.69–1.17); Q4: OR = 0.80 (0.65–0.98); *p* trend = 0.1 ***nvAMD*** *Unadjusted OR* Q2: OR = 0.88 (0.55–1.39); Q3: OR = 0.62 (0.33–1.16); Q4: OR = 0.52 (0.29–0.93); *p* trend = 0.03 *Adjusted OR* Q2: OR = 0.83 (0.55–1.26); Q3: OR = 0.62 (0.39–1.00); Q4: OR = 0.53 (0.27–1.04); *p* trend = 0.01
**Case–control studies**				
Nunes et al. (2018) [35]	Validated FFQ (86 items) mediSCORE (0–9); high adherence = ≥6	AMD graded according to the Rotterdam Classification for AMD	High mediSCORE vs. prevalence of AMD	Prevalence of AMD OR = 0.73 (0.58–0.93, *p* = 0.009))
Raimundo et al. (2018) [36]	Validated FFQ (86 items) mediSCORE (0–9); high adherence = ≥6	AMD graded according to the Rotterdam Classification for AMD	High mediSCORE (≥6) vs. prevalence of AMD (unadjusted and adjusted for age, gender and calories consumption)	Prevalence of AMD *Unadjusted:* 38.4% vs. 50.5%, *p* = 0.041, OR: 0.62 (0.38–0.97) *Adjusted:* OR: 0.63 (0.41–0.98), *p* = 0.043
Barreto, et al. (2023) [37]	Validated FFQ (86 items) mediSCORE (0–9); high adherence = ≥6 Two groups: low MD adherence (0–3) or medium-high MD adherence (4–9)	AMD graded according to the Rotterdam Classification for AMD	High mediSCORE (≥6) vs. low mediSCORE (<6) (adjusted for age, sex, physical exercise, and smoking)	Prevalence of AMD OR = 0.406 (0.226–0.728, *p* = 0.002)
**Prospective cohort studies**				
Merle et al. (2015) [14]	Validated, self-administered, 90-item, semiquantitative FFQ at baseline mediSCORE (0–9) - Low MD adherence was defined as mediSCORE ≤3 - Medium MD adherence was defined as mediSCORE = 4–5 - High MD adherence was defined as mediSCORE ≥6	CARMS Progression was defined as either eye progressing from no, early, or intermediate AMD at baseline to advanced disease (either GA or nvAMD)	Low MD adherence was defined as reference Two models 1. Adjusted for age, sex, AREDS treatment, AMD grade at baseline for both eyes, and total energy intake 2. Adjusted for age, sex, AREDS treatment, AMD grade at baseline for both eyes, and total energy intake, educational level, smoking, BMI, supplement use, and 10 genetic variants [CFH rs1061170 (Y402H), CFH rs1410996, CFHrs121913059 (R1210C), ARMS2/HTRA1 rs10490924, C2 rs9332739 (E318D), CFB rs641153 (R32Q), C3 rs2230199 (R102G), C3 rs147859257 (K155Q), COL8A1 rs13095226, and RAD51B rs8017304]	High mediSCORE (≥6) vs. low mediSCORE (≤3) *Model 1*: HR = 0.91 (0.77–1.07) *Model 2:* HR = 0.92 (0.78–1.07) Intermediate mediSCORE (4–5) vs. low mediSCORE score (≤3) *Model 1:* 0.74 (0.61–0.90) *Model 2:* 0.74 (0.61–0.91)
Merle et al. (2019) [33]	*RS-I:* 170-item validated semiquantitative FFQ at baseline *Alienor:* 40-item validated FFQ at baseline and a 24 h dietary recall mediSCORE (0–9) - Low MD adherence was defined as ≤3 - Medium MD adherence was defined as (4–5) - High MD adherence was defined as (≥6)	AMD graded based on the Wisconsin Age-Related System (RS-I) and the ICS (Alienor) Incidence of advanced AMD was defined as the participant progressing from no or early AMD at baseline to advanced AMD (either neovascular or atrophic)	Low MD adherence was defined as reference Two models 1. Unadjusted 2. Adjusted for gender, total energy intake, age-related macular degeneration grade at baseline, education, body mass index, smoking, supplement use of multivitamins or minerals, and presence of diabetes and hypercholesterolemia.	***MODEL 1*** *RS-1:* 1. Medium vs. low MD adherence: HR= 0.69 (0.46–1.03) 2. High vs. low MD adherence: HR = 0.56 (0.33–0.96) *Alienor:* 1. Medium vs. low MD adherence: HR = 0.80 (0.39–1.63) 2. High vs. low MD adherence: HR = 0.48 (0.18–1.26) *Overall:* 1. Medium vs. low MD adherence: HR = 0.71 (0.50–1.00) 2. High vs. low MD adherence: HR = 0.53 (0.33–0.84) ***MODEL 2*** *RS-1:* 1. Medium vs. low MD adherence: HR = 0.70 (0.46–1.06) 2. High vs. low MD adherence: HR = 0.69 (0.40–1.20) *Alienor:* 1. Medium vs. low MD adherence: HR = 0.83 (0.38–1.80) 2. High vs. low MD adherence: HR = 0.52 (0.19–1.40) *Overall:* 1. Medium vs. low MD adherence: HR = 0.70 (0.49–1.01) 2.High vs. low MD adherence: HR = 0.59 (0.37–0.95)
Merle et al. (2020) [38]	Validated, self-administered, semiquantitative FFQ (90 items) at AREDS baseline aMED score (0–9) - Low MD adherence was defined as ≤3 - Medium MD adherence was defined as (4–5) - High MD adherence was defined as (≥6)	Eye-specific progression of maximum drusen size was defined as one eye advancing at least two grades during the study period	Medium-high MD adherence (≥4) versus low MD adherence (≤3)	HR = 0.83 (0.68–0.99), *p* = 0.049

OR: odds ratio; HR: hazard ratio; FFQ: Food Frequency Questionnaire; MDScore: Mediterranean diet score; MD: Mediterranean diet; AMD: age-related macular degeneration; nvAMD: neovascular AMD; BMI: body mass index; ICS: International Classification System; mediSCORE: adherence to the Mediterranean diet score; CARMS: AMD graded according to Clinical Age-Related Maculopathy Staging; GA: geographic atrophy; AREDS: Age-Related Eye Disease Study; RS-I: Rotterdam Study I; Alienor: Antioxydants, Lipides Essentiels, Nutrition et Maladies Oculaires (Alienor) Study.

## Data Availability

All data produced and examined in this study are fully available within this published article and the Supplementary Materials.

## Supplementary Materials

The following supporting information can be downloaded at: https://www.mdpi.com/article/10.3390/nu17061037/s1, Table S1: Queries used for searching MEDLINE/PubMed, Web of Science and Scopus; Table S2: Reports excluded at the screening phase with reasons for exclusion; Figure S1: Baujat plot for cross-sectional studies assessing study contribution to heterogeneity; Figure S2: Baujat plot for case-control studies assessing study contribution to heterogeneity; Figure S3: Baujat plot for prospective cohort studies assessing study contribution to heterogeneity; Figure S4: Funnel plot assessing publication bias in the meta-analysis of cross-sectional studies; Figure S5: Funnel plot assessing publication bias in the meta-analysis of case-control studies; Figure S6: Funnel plot assessing publication bias in the meta-analysis of prospective cohort studies.
