# Supplementary material for: Mediterranean Diet on Development and Progression of Age-Related Macular Degeneration: Systematic Review and Meta-Analysis of Observational Studies
Source: Nutrients. 2025 Mar 15;17(6):1037. doi: 10.3390/nu17061037 (PMC11944936; doi:10.3390/nu17061037)

Supplementary Material

Table S1. Queries used for searching MEDLINE/PubMed, Web of Science and Scopus.

Search	Query	Items found
	**MEDLINE/PubMed Search Strategy**	
**#1**	**1st Concept - terms relating to age-related macular degeneration**<br><br>("Macular Degeneration"[MeSH] OR "age-related macular degeneration" OR "AMD" OR "age related macular degeneration" OR "age-related maculopathy" OR "maculopathy, age-related" OR "maculopathy" OR "Retinal Diseases"[MeSH] OR "retinal degeneration" OR "retina degeneration" OR "age-related retinal degeneration" OR "retinal diseases" OR "retinal disease" OR "macular diseases" OR "macular disease" OR "retina")	275,953
**#2**	**2nd Concept – terms relating to Mediterranean diet**<br><br>("Diet, Mediterranean"[MeSH] OR "Mediterranean diet" OR "diet, Mediterranean" OR "med diet" OR "mediterranean eating pattern" OR "mediterranean dietary" OR "mediterranean dietary pattern" OR "mediterranean dietary habits" OR "traditional mediterranean diet" OR "mediterranean lifestyle" OR "mediterranean style diet" OR "mediterranean-style diet" OR "mediterranean diet score" OR "mediterranean diet index" OR "mediterranean cuisine" OR "mediterranean nutrition" OR "mediterranean food pattern" OR "mediterranean nutrition" OR "Diet, Vegetarian"[MeSH] OR "plant-based diet" OR "vegetarian" OR "dietary lifestyle" OR "dietary pattern" OR “healthy lifestyles” OR “healthy lifestyle”)	36,918
**#3**	#1 AND #2	149

	Web of Science Search Strategy	
#1	**1st Concept - terms relating to age-related macular degeneration**<br>("Macular Degeneration" OR "age-related macular degeneration" OR "AMD" OR "age related macular degeneration" OR "age-related maculopathy" OR "maculopathy, age-related" OR "maculopathy" OR "retinal degeneration" OR "retina degeneration" OR "age-related retinal degeneration" OR "retinal diseases" OR "retinal disease" OR "macular diseases" OR "macular disease" OR "retina") (All Fields)	**213,885**
#2	**2nd Concept – terms relating to Mediterranean diet**<br>("Mediterranean diet" OR "diet, Mediterranean" OR "med diet" OR "mediterranean eating pattern" OR "mediterranean dietary" OR "mediterranean dietary pattern" OR "mediterranean dietary habits" OR "traditional mediterranean diet" OR "mediterranean lifestyle" OR "mediterranean style diet" OR "mediterranean-style diet" OR "mediterranean diet score" OR "mediterranean diet index" OR "mediterranean cuisine" OR "mediterranean nutrition" OR "plant-based diet" OR "vegetarian" OR "dietary lifestyle" OR "dietary pattern" OR "healthy lifestyle" OR "healthy lifestyles") (All Fields)	**45,029**
#3	**#1 AND #2**	**202**
	**Scopus Search Strategy**	
#1	**1st Concept - terms relating to age-related macular degeneration**<br>TITLE-ABS-KEY ("Macular Degeneration" OR "age-related macular degeneration" OR "AMD" OR "age related macular degeneration" OR "age-related maculopathy" OR "maculopathy, age-related" OR "maculopathy" OR "retinal degeneration" OR "retina degeneration" OR "age-related retinal degeneration" OR "retinal diseases" OR "retinal disease" OR "macular diseases" OR "macular disease" OR "retina")	358,482
#2	**2nd Concept – terms relating to Mediterranean diet**<br>TITLE-ABS-KEY ("Mediterranean diet" OR "diet, Mediterranean" OR "med diet" OR "mediterranean eating pattern" OR "mediterranean dietary" OR "mediterranean dietary pattern" OR "mediterranean dietary habits" OR "traditional mediterranean diet" OR "mediterranean lifestyle" OR "mediterranean style diet" OR "mediterranean-style diet" OR "mediterranean diet score" OR "mediterranean diet index" OR "mediterranean cuisine" OR "mediterranean nutrition" OR "plant-based diet" OR "vegetarian" OR "dietary lifestyle" OR "dietary pattern" OR "healthy lifestyle" OR "healthy lifestyles")	72,385
#3	**#1 AND #2**	**271**

Table S2. Reports excluded at the screening phase with reasons for exclusion.

Reason for exclusion	Study (author; year of publication)	Title	Journal
**Wrong publication type**	Tiarnan D.L. Keenan, et al.; 2023	Geographic Atrophy in Age-Related Macular Degeneration: A Tale of Two Stages	Ophthalmology Science
	S. A. Sondel, et al.; 2010	Relationship of Healthy Lifestyles to the Prevalence of Intermediate Age-Related Macular Degeneration (AMD) in the Carotenoids in Age-Related Eye Diseases Study (CAREDS), an Ancillary Study of the Women's Health Initiative Observational Study (WHIOS)	Investigative Ophthalmology & Visual Science
	Benedicte MJ Merle, et al.; 2015	Mediterranean diet, genetic susceptibility and progression to advanced macular degeneration.	Investigative Ophthalmology & Visual Science
	M. Fleckenstein, et al.; 2019	Altersabhängige Makuladegeneration	Ophthalmologe
**Wrong diet**	Shruti Dighe, et al.; 202015/03/2025 19:51:00	Diet patterns and the incidence of age-related macular degeneration in the Atherosclerosis Risk in Communities (ARIC) study	British Journal of Ophthalmology
	Yanhui Lin, et al.; 2022	The frequency of early age-related macular degeneration and its relationship with dietary pattern in Hunan, China: a cross-sectional study	BMC Ophthalmology

	Stefano Piermarocchi, et al.; 2016	Risk Factors and Age-Related Macular Degeneration in a Mediterranean-Basin Population: The PAMDI (Prevalence of Age-Related Macular Degeneration in Italy) Study - Report 2	Ophthalmic Research
	Martha P Montgomery, et al.; 2010	Overall Diet Quality and Age-Related Macular Degeneration	Ophthalmic Epidemiology
	Kyung Tae Kang, et al.; 2019	Dietary Patterns and Age-Related Macular Degeneration in Korea: The Korea National Health and Nutrition Examination Survey	Scientific Reports
**Wrong outcome**	Geoffrey K Broadhead, et al.; 2023	Association of Dietary Nitrate and a Mediterranean Diet With Age-Related Macular Degeneration Among US Adults: The Age-Related Eye Disease Study (AREDS) and AREDS2	JAMA Ophthalmology
	Nadia San Onofre Bernat, et al.; 2022	Health Determinants Associated with the Mediterranean Diet: A Cross-Sectional Study	Nutrients
**Duplicate**	Bénédicte M J Merle et al.; 2019	Mediterranean Diet and Incidence of Advanced Age-Related Macular Degeneration: The EYE-RISK Consortium	Ophtahlmology Journal
**Wrong study design**	Agrón, et al.; 2022	Adherence to a Mediterranean Diet and Geographic Atrophy Enlargement Rate:Age-Related Eye Disease Study 2 Report 29	Ophthalmology Retina
	Keenan et al.; 2020	Adherence to the Mediterranean Diet and Progression to Late Age-Related Macular Degeneration in the Age-Related Eye Disease Studies 1 and 2	Ophthalmology Journal
	Ajana et al.; 2021	Predicting Progression to Advanced Age-Related Macular Degeneration from Clinical, Genetic, and Lifestyle Factors Using Machine Learning	

Figure S1. Baujat plot for cross-sectional studies assessing study contribution to heterogeneity.

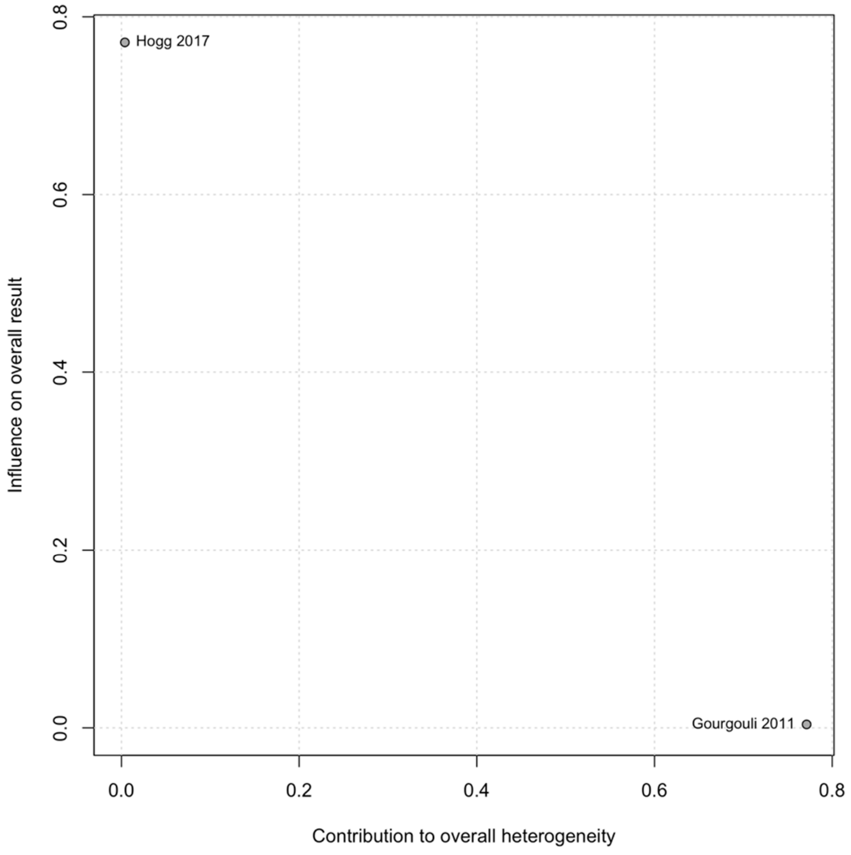

Figure S2. Baujat plot for case-control studies assessing study contribution to heterogeneity.

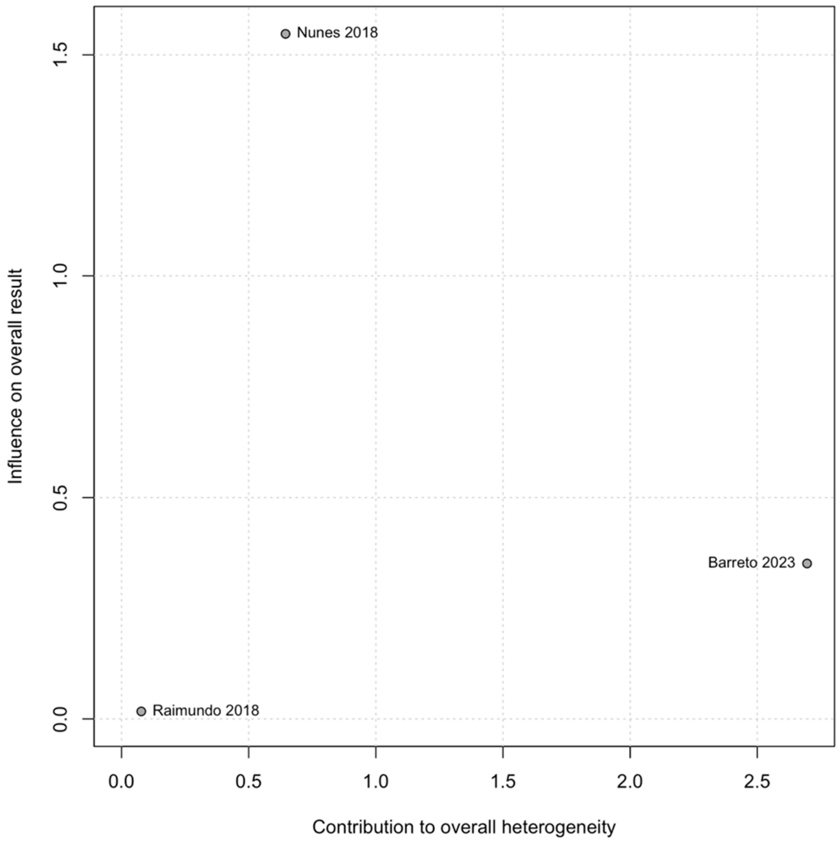

Figure S3. Baujat plot for prospective cohort studies assessing study contribution to heterogeneity.

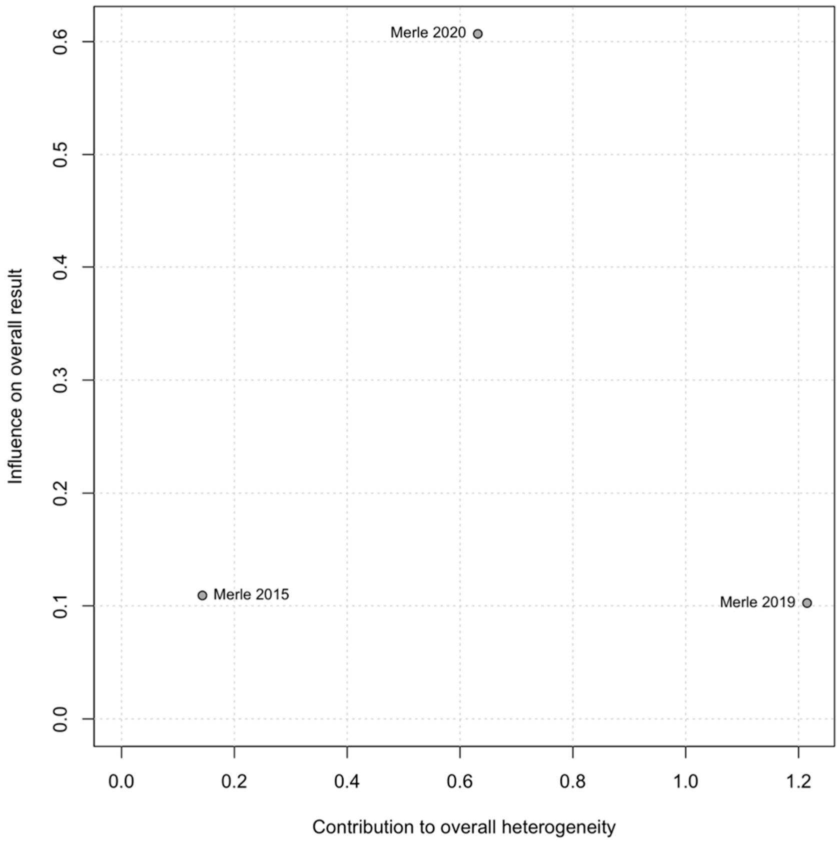

Figure S4. Funnel plot assessing publication bias in the meta-analysis of cross-sectional studies.

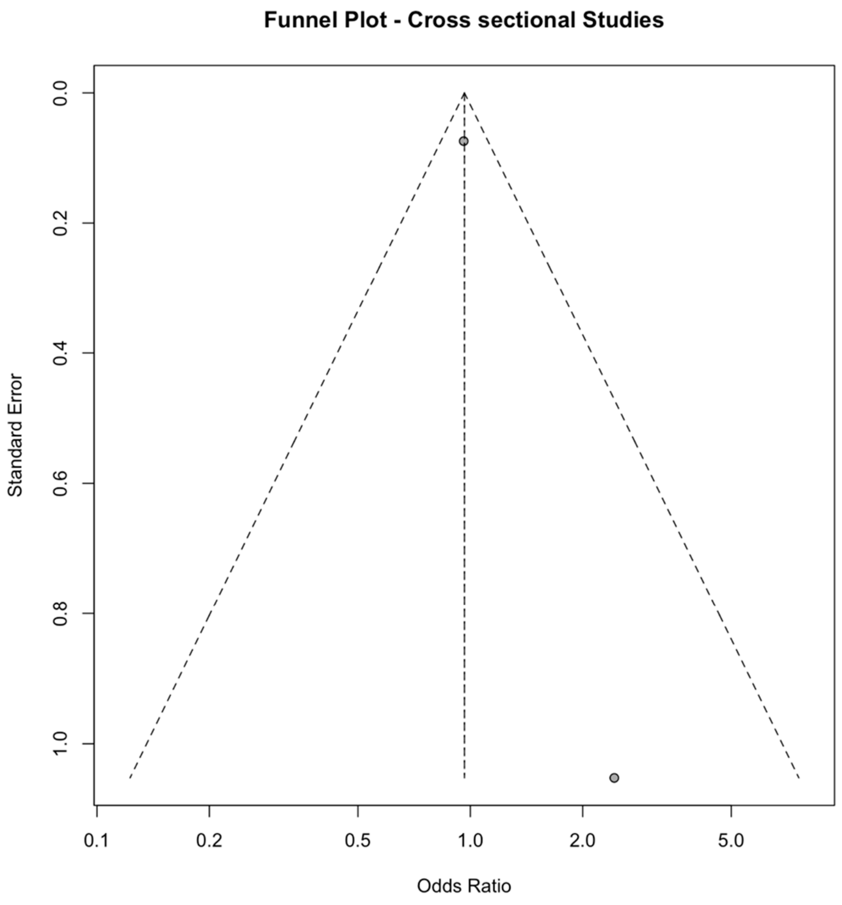

Figure S5. Funnel plot assessing publication bias in the meta-analysis of case-control studies.

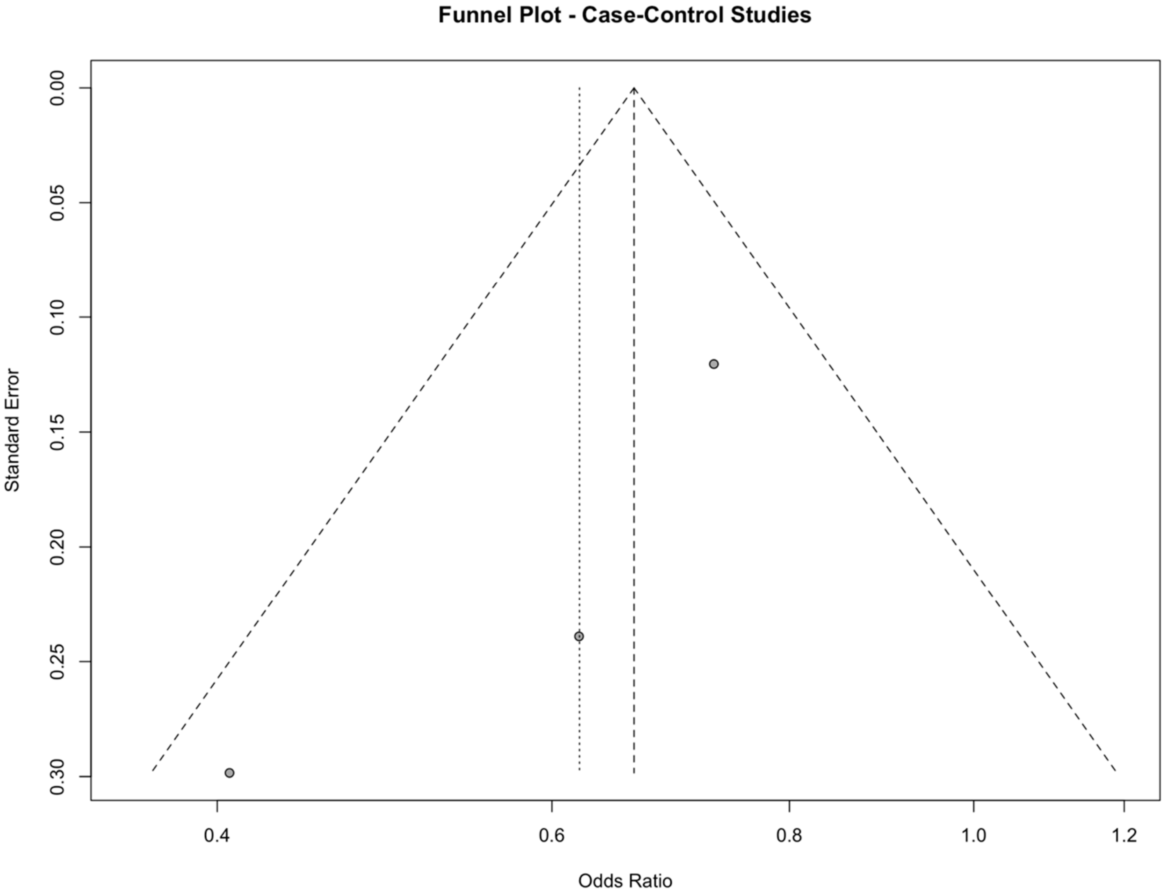

Figure S6. Funnel plot assessing publication bias in the meta-analysis of prospective cohort studies.

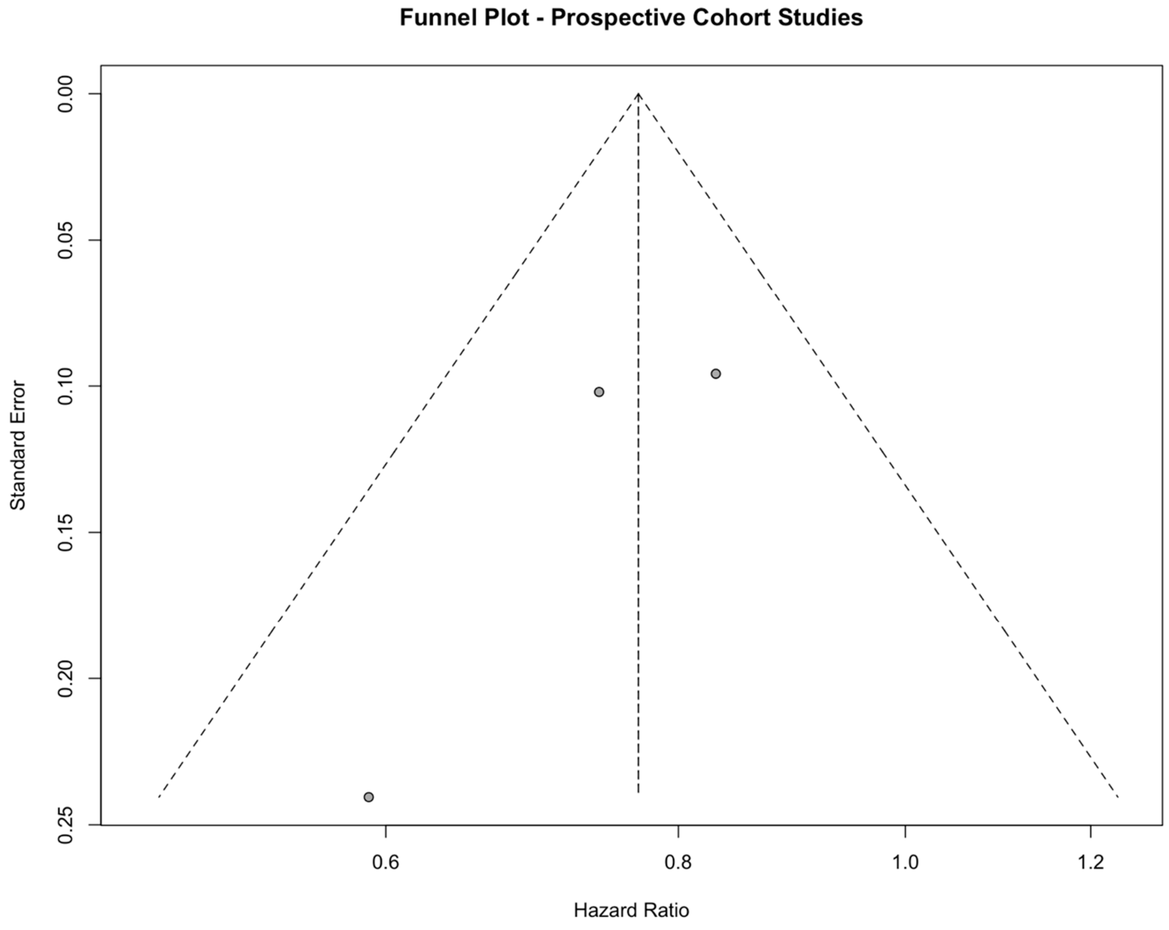

Supplement: Supplementary file 1 [file nutrients-17-01037-s001.zip › nutrients-3507029-supplementary.pdf]
